# Development and characterization of a long-acting allosteric growth hormone receptor antagonist for acromegaly

**DOI:** 10.1210/endocr/bqag090

**Published:** 2026-08-07

**Authors:** Katherine Kurylo, Page R Bouchard, Mark N Milton, Peter F Moesta, Laura E Dichtel, Jesper Gromada, Vyas Ramanan, Thomas Vincent, Melinda Smith, Chew Shun Chang, Jack McGuire, Richard Shimkets, Mark P Joing, Joshua Lehrer-Graiwer, Beryl B Cummings, Ethan J Weiss

**Affiliations:** Marea Therapeutics, South San Francisco, CA 94080, USA; Marea Therapeutics, South San Francisco, CA 94080, USA; Marea Therapeutics, South San Francisco, CA 94080, USA; Marea Therapeutics, South San Francisco, CA 94080, USA; Marea Therapeutics, South San Francisco, CA 94080, USA; Mass General Brigham Innovation Fellows Program, Massachusetts General Hospital/Third Rock Ventures, Boston, MA 02114, USA; Marea Therapeutics, South San Francisco, CA 94080, USA; Marea Therapeutics, South San Francisco, CA 94080, USA; Alloy Therapeutics, Waltham, MA 02451, USA; Alloy Therapeutics, Waltham, MA 02451, USA; Alloy Therapeutics, Waltham, MA 02451, USA; Alloy Therapeutics, Waltham, MA 02451, USA; Alloy Therapeutics, Waltham, MA 02451, USA; Marea Therapeutics, South San Francisco, CA 94080, USA; Marea Therapeutics, South San Francisco, CA 94080, USA; Marea Therapeutics, South San Francisco, CA 94080, USA; Marea Therapeutics, South San Francisco, CA 94080, USA

**Keywords:** acromegaly, growth hormone receptor antagonist, MAR002, pegvisomant, IGF-1

## Abstract

Acromegaly is a rare disease caused by growth hormone (GH) hypersecretion from a pituitary adenoma. Pegvisomant is the only approved GH receptor antagonist (GHRA) and is administered via daily subcutaneous injections, which limits real-world effectiveness. Here, we report the discovery and characterization of MAR002, a novel, half-life extended monoclonal antibody GHRA. MAR002 was identified by immunizing transgenic mice with the extracellular domain of human and cynomolgus monkey GH receptor (GHR) and engineered with Fc modifications to limit effector function and extend serum half-life. MAR002 was benchmarked against pegvisomant in biophysical assays, cellular signaling inhibition studies, and a head-to-head pharmacokinetic/pharmacodynamic study in cynomolgus monkeys. MAR002 exhibited higher binding affinity to GHR and >100-fold more potent inhibition of GH-induced GHR signaling than pegvisomant (human half-maximal inhibitory concentration [IC_50_] 1.05 nM vs 122 nM). Epitope binning demonstrated simultaneous binding of MAR002 and GH to GHR, consistent with an allosteric, noncompetitive mechanism; notably, MAR002 inhibitory activity was maintained at supraphysiological GH concentrations, whereas pegvisomant activity was competitively reduced. In cynomolgus monkeys, a single 10 mg/kg intravenous dose of MAR002 sustained ≥50% IGF-1 suppression for 36 days compared with 15 days for an equimolar pegvisomant dose, with comparable maximal lowering (∼80%). These findings support clinical evaluation of MAR002 as a next-generation long-acting GHRA for acromegaly, with potential for reduced dosing frequency and more consistent suppression of the GH–IGF-1 axis.

Acromegaly is a rare endocrine disorder caused by chronic growth hormone (GH) excess, most commonly due to a GH-secreting pituitary adenoma, resulting in elevated insulin-like growth factor-1 (IGF-1), somatic overgrowth, and multisystem complications. Treatment options include surgery, radiation, and medical therapies directed against the somatotroph adenoma or GH action. Normalization of IGF-1 is the primary goal of medical therapy, with most patients requiring lifelong treatment (1, 2).

Current medical options in acromegaly have notable limitations. Somatostatin receptor ligands (SRLs) achieve IGF-1 normalization in only 30% to 50% of patients and are associated with adverse effects, including gastrointestinal intolerance and impaired glucose metabolism (2-6). GH receptor (GHR) antagonism is an attractive alternative therapeutic strategy as it directly inhibits GH action at its receptor, reduces IGF-1 production, and improves insulin sensitivity (4). The only approved GHR antagonist (GHRA), pegvisomant, has been shown to normalize IGF-1 in ∼90% of patients in clinical trials (2, 7-10). However, its high treatment burden of daily injections impacts patient adherence and real-world efficacy (1, 2, 8, 11-13). Consequently, there remains a significant unmet need in acromegaly for medical therapies that combine improved efficacy with a reduced treatment burden.

Here, we report the discovery and characterization of MAR002, a novel monoclonal antibody GHRA. MAR002 was characterized with respect to its ligand-binding properties, in vitro activity, and in vivo PK and PD properties in direct comparison with pegvisomant to highlight the attributes that support its continued clinical development as a potential long-acting therapy for acromegaly.

## Materials and methods

### Discovery and engineering of MAR002

MAR002 was discovered using the proprietary ATX-Gx™ humanized transgenic mouse platform (Alloy Therapeutics, Waltham, MA, USA). Ten mice were immunized with commercially available recombinant extracellular domain (ECD) of human GHR (Cat# GHR-H5222) and cynomolgus monkey GHR (Cat# GHR-C52H1) protein (ACROBiosystems, Newark, DE, USA). Both human and cynomolgus monkey GHR ECDs were used for immunization to generate antibodies with cross-reactivity to both species, enabling comprehensive in vitro characterization and supporting subsequent in vivo evaluation in cynomolgus monkeys. Anti-GHR antibody titers in serum were determined by direct enzyme-linked immunosorbent assay (ELISA), in which human or cynomolgus monkey GHR ECD (1 µg/mL) was immobilized overnight at 4 °C on the plate surface (Corning, Corning, NY, USA; Cat# 3690). To isolate antigen-specific B cells for variable heavy and variable light gene sequencing, single-cell suspensions of splenocytes and lymph node lymphocytes were depleted of T cells and IgM^+^ B cells. Cells were then incubated with biotinylated human GHR ECD (ACROBiosystems, Cat# GHR-H8222) and Dynabeads™ M-280 Streptavidin magnetic beads (Thermo Fisher Scientific, Waltham, MA, USA; Cat# 11205D), and antigen-specific B cells were isolated using a magnet and sequenced. The resulting next-generation sequencing sequence data were mined to generate a diverse panel of anti-GHR antibodies.

Antibody binding to GHR was screened using both recombinant and cell-based formats. Interaction with the recombinant GHR ECD was quantified by surface plasmon resonance (SPR) on a Carterra LSA or Biacore T200 platform. To confirm binding to native, full-length receptor, antibodies were evaluated by flow cytometry using suspension-adapted HEK293 cells (FreeStyle™ 293-F; Thermo Fisher Scientific; RRID:CVCL_D603) expressing human GHR (cell line generation described below). Cells were seeded at 2 × 10^5^ per well in polypropylene 96-well plates and incubated with each antibody at 7 concentrations ranging from 0.04 to 30 nM (50 µL/well) on ice for 1 hour. Following 2 washes with fluorescence-activated cell sorting (FACS) buffer (eBioscience, San Diego, CA, USA; Cat# 00-4222-26), a fluorescently labeled antihuman secondary antibody (1:800 dilution; Jackson ImmunoResearch Laboratories, West Grove, PA, USA; Cat# 709-136-149; RRID:AB_2340526) was added and incubated on ice for 30 minutes. Cells were washed, resuspended in FACS buffer containing 1% paraformaldehyde, and analyzed on a MACSQuant flow cytometer (Miltenyi Biotec, Bergisch Gladbach, Germany). Data were analyzed using FlowJo software (version 10; RRID:SCR_008520). Antibodies confirmed to bind both human and cynomolgus monkey GHR ECD and full-length GHR were further characterized to confirm functional activity with methods described below. The precursor to MAR002 was selected for further development based on those results and was subsequently engineered as a human IgG1 with LALA (L234A/L235A) mutations to limit Fc effector function and the LS (M428L/N434S) mutations to extend serum half-life by enhancing binding to the neonatal Fc receptor (FcRn) (14-16).

### Binding kinetics and epitope binning assays

Binding kinetics and epitope binning were analyzed by SPR. For binding kinetics, assays were conducted at 25 °C at a flow rate of 30 µL/min using 1× HBS-EP^+^ pH 7.4 buffer diluted with deionized water. An anti-histidine antibody (His Capture Kit; Cytiva, Marlborough, MA, USA; Cat# 28995056; RRID:AB_3750253) was immobilized on a sensor chip via amine coupling, followed by capture of His-tagged human, cynomolgus monkey, or rat GHR ECD (ACROBiosystems, Cat# GHR-H5222, GHR-C52H1, and GHR-R52H8, respectively) or mouse GHR ECD (Sino Biological, Wayne, PA, USA; Cat# 50043-M08H) with a 60-second contact time. Following receptor capture, analytes were injected with a 120-second association phase and a 10-minute dissociation phase. GH (Charles River Laboratories, Wilmington, MA, USA), pegvisomant (Somavert; Pfizer Inc., New York, NY, USA), and MAR002 were all tested using a 6-point series with a top concentration of 200 nM, 2 µM, and 500 nM, respectively, and 1:3 serial dilutions for each. Binding kinetic experiments were performed using human and cynomolgus monkey GHR ECD with an exon 3 deletion (d3-GHR; Charles River Laboratories) and the human prolactin receptor (PRLR; ACROBiosystems; Cat# PRR-H52Ha) using methodologies similar to those described above. These experiments assessed MAR002 binding to a prevalent GHR isoform (d3-GHR) and selectivity against PRLR, a structurally related class I cytokine receptor to which GH is also known to bind.

To confirm enhanced FcRn binding conferred by the LS mutation in the Fc region of MAR002, binding kinetics of MAR002 to human and cynomolgus monkey FcRn were assessed by SPR using methods similar to those employed for GHR binding, with the additional use of 1× PBS-P+ 0.05% P20 pH 6.0 diluted with deionized H_2_O to assess pH-dependent binding. Captured human or cynomolgus monkey FcRn-his (ACROBiosystems; Cat# FCM-H5286 and FCM-C5284, respectively) were used as ligands with a 120-second dissociation time. The precursor to MAR002, which lacks the LS mutation, was used as a comparator. For all binding kinetic experiments, association (*k_a_*) and dissociation (*k_d_*) rates were measured and used to calculate the equilibrium dissociation constants (KD) with the equation KD = *k_d_*/*k_a_*. Data were buffer and reference-surface subtracted and fitted with a 1:1 Langmuir binding equation.

SPR epitope binning assays were performed to determine whether MAR002 and GH can bind simultaneously to the GHR. Assays were conducted using a methodology similar to the binding kinetic experiments, with an anti-histidine antibody immobilized on a sensor chip via amine coupling and subsequent capture of His-tagged human or cynomolgus monkey GHR ECD with a 60-second contact time. Following receptor capture, a dual-injection protocol was used in which GH was first injected to form a GHR-GH complex with a 120-second contact time, immediately followed by a second injection of either MAR002 or GH with a 150-second contact time to assess binding to the preformed complex. Assays were performed in 1× HBS-EP^+^ buffer diluted with deionized water at a flow rate of 10 µL/min. Control experiments were conducted in the absence of GH injection to determine the magnitude of MAR002 binding to GHR ECD alone. GH was used as the sole reference ligand to assess competitive binding in these assays, as pegvisomant is a GH analog that occupies the same canonical GHR binding site (7).

### Hydrogen–deuterium exchange mass spectrometry

The binding epitope of MAR002 on the human GHR ECD was characterized by hydrogen–deuterium exchange mass spectrometry (HDX-MS) at Rapid Novor (Kitchener, ON, Canada). GHR ECD (6 µM) was analyzed in the absence and presence of MAR002 (8 µM). Samples were diluted into a D_2_O-based phosphate buffer (pD 7.4; deuterium oxide, 99.9% D; Canadian Life Science, Toronto, ON, Canada; Cat# NMR-DTOX) and incubated for 2, 10, and 60 minutes to initiate deuterium exchange. Reactions were quenched by reducing the pH to 2.4 with a buffer containing 4 M urea and 200 mM tris(2-carboxyethyl)phosphine hydrochloride (TCEP-HCl; Thermo Fisher Scientific; Cat# 20490). Quenched samples were subjected to automated online proteolysis using pepsin digestion and PNGase Rc deglycosylation, followed by ultra-performance liquid chromatography–mass spectrometry on a Waters Cyclic IMS mass spectrometer (Waters Corporation, Milford, MA, USA) equipped with a Trajan Leap HDX automated sample handler. Peptide identification was performed using ProteinLynx Global Server (Waters Corporation, version 3.0.3) and deuterium uptake analysis was performed using DynamX (Waters Corporation, version 3.0.0). Peptide-level deuterium incorporation was compared between unbound and MAR002-bound states, and regions exhibiting a reduction in deuterium uptake >5% were considered protected and indicative of epitope engagement. Protected regions were mapped onto the GHR ECD crystal structure (Protein Data Bank [PDB]: 3HHR) for structural visualization. Experiments were performed in at least triplicate.

### Cell line generation and in vitro GHR inhibition assays

Suspension-adapted HEK293 cells (FreeStyle™ 293-F Cells; Thermo Fisher Scientific) stably expressing full-length human or cynomolgus monkey GHR were used for all functional assays. This overexpression system provided the flexibility to assess MAR002 potency across multiple GHR species orthologs in an identical assay format, enabling direct comparison of MAR002 activity at human and cynomolgus monkey GHR. Stable expression was achieved by transducing FreeStyle 293-F cells at a multiplicity of infection of 10 with lentiviral vectors encoding GHR and a puromycin resistance cassette (Origene Technologies Inc., Rockville, MD, USA). Culture medium (FreeStyle™ 293 Expression Medium; Thermo Fisher Scientific; Cat# 12338018) was replaced 24 hours post-transduction to remove residual lentiviral particles. After an additional 72 hours, cells were transferred to selection medium containing puromycin (1 µg/mL; Thermo Fisher Scientific; Cat# A1113803) to eliminate nontransduced cells. Cells were maintained under selection for ∼1 week, after which stable expression of functional GHR was confirmed by flow cytometry using methods analogs to those described above, and functional assays (see below) relative to parental, nontransduced FreeStyle 293-F cells.

Functional activity was assessed by measuring phosphorylation of Signal Transducer and Activator of Transcription 5 (STAT5; pSTAT5 [Tyr694]), a downstream readout of GHR signaling, by using a homogeneous time-resolved fluorescence (HTRF) assay kit (Revvity, Waltham, MA, USA; Cat# 64AT5PEG), according to the manufacturer's instructions. The HTRF signal was measured using a CLARIOstar Plus plate reader (BMG Labtech, Ortenberg, Germany). The specificity of the pSTAT5 functional assay was established during assay validation. No detectable pSTAT5 signal was observed in parental FreeStyle 293-F cells lacking GHR expression following stimulation with 30 nM GH, confirming that STAT5 activation in the engineered cell line was GHR-dependent.

To evaluate inhibitory activity, cells were preincubated with serial dilutions of MAR002 (0.2-75 nM) or pegvisomant (1-1000 nM) for 30 minutes prior to stimulation with 5 nM human GH, corresponding to the predetermined EC_90_ derived from prior GH dose-response experiments. GH-induced signaling was quantified by measuring pSTAT5 levels using the same fluorescence (HTRF) assay kit described above. The percent inhibition was calculated by normalization to a no-GH/no-antagonist negative control and a GH-only positive control. To further confirm that signaling inhibition was attributable to the functional activity of MAR002 rather than nonspecific antibody effects, a GHR binding, noninhibitory antibody identified from the same discovery antibody screening was used as a negative control. This control demonstrated that receptor occupancy by a nonfunctional antibody was insufficient to alter pSTAT5 signaling.

Half-maximal inhibitory concentrations (IC_50_) were determined by fitting dose-response curves using a 4-parameter logistic regression model in GraphPad Prism (GraphPad Software, version 9; RRID:SCR_002798). Additional assays were performed using similar methods with increasing GH concentrations (0.06-1215 nM) to assess activity under conditions of GH excess and receptor occupancy.

To assess the potential contribution of avidity to the observed potency, a monovalent fragment antigen-binding (Fab) of MAR002 was generated by Alloy Therapeutics, expressed directly from a corresponding vector, and purified using CaptureSelect™ CH1-XL Affinity Matrix (Thermo Fisher Scientific; Cat# 1943462250). Fab potency was compared with the full-length IgG1 format in the pSTAT5 HTRF assay with the methodology described above, using a single concentration of 30 nM for each molecule against the EC_90_ concentration of GH (5 nM).

### In vivo pharmacokinetic and pharmacodynamic study

MAR002 was evaluated in an exploratory, non-Good Laboratory Practice (non-GLP), nonterminal cynomolgus monkey study with pegvisomant used as a comparator compound. All animal procedures were conducted in accordance with the guidelines of the Institutional Animal Care and Use Committee (protocol no. 22-03). Animals were single or socially housed in stainless steel cages with an automatic watering valve and provided Monkey Diet 5038 (Lab Diet) daily. Filtered tap water was provided ad libitum. Environmental conditions were maintained at a temperature of 64-84°F, humidity of 30-70%, and a 12-hour light/dark cycle. The study was conducted exclusively in male Mauritian cynomolgus monkeys (4-6 years of age), obtained from Bioculture Group (Rivière des Anguilles, Mauritius) to minimize biological variability and reduce the number of animals required for this exploratory pharmacodynamic (PD) evaluation. The animals were maintained according to the NIH Guide for the Care and Use of Laboratory Animals (17). This single-sex design avoids the confounding influence of cyclical endogenous sex steroids in females, which are known to produce sexual dimorphism in GH secretion and GH–IGF-1 axis regulation, including differences in pulsatile GH secretion patterns (18, 19). Evaluation of MAR002 in both sexes is planned for subsequent formal toxicology studies.

Male cynomolgus monkeys (*n* = 3 per group) were administered a single intravenous (IV) dose of either MAR002 (10 mg/kg) or an equimolar dose of pegvisomant (3 mg/kg). The equimolar pegvisomant dose, calculated using the molecular weight of the PEGylated form of pegvisomant (∼45 kDa) relative to MAR002 (∼150 kDa), was selected to enable direct comparison of PD activity between the 2 molecules. The IV route was selected to ensure maximal bioavailability and to enable accurate characterization of pharmacokinetic/pharmacodynamic (PK/PD) properties of MAR002 independent of absorption kinetics. Due to the exploratory nature of the study, no formal power calculations were performed. A group size of 3 animals per treatment is consistent with established practice for exploratory, non-GLP PD studies in nonhuman primates and was considered sufficient to characterize the primary PK/PD endpoints given the magnitude of effect anticipated based on the in vitro potency data. Blood samples were collected into serum separator tubes on Day −12 and Day −7, at 1, 6 , and 24 hours postdose, and on Days 3, 5, 7, 10, 14, 18, 21, 28, 39, 46, and 53. Samples at Days 46 and 53 were collected only from animals receiving MAR002 to capture the extended PK/PD profile of the antibody. Serum was isolated and stored at −80 °C until analysis. The dosing and sample collection was performed by JOINN Laboratories CA, Inc. (Biomere), Richmond, CA, USA. Baseline serum IGF-1 concentration was defined as the mean of the 2 predose samples collected on Day −12 and Day −7. Postdose IGF-1 values were expressed as a percentage of baseline.

MAR002 concentrations were measured using a human IgG1 ELISA (Cayman Chemical, Washtenaw County, MI, USA; Cat# 500910; RRID:AB_3750265). Pegvisomant concentrations were measured using a PEGylated protein ELISA (Abcam, Waltham, MA, USA; Cat# ab133065; RRID:AB_3750262). Total IGF-1 and GH were measured using Quantikine ELISA kits (R&D Systems, Minneapolis, MN, USA; IGF-1: Cat# DG100B; RRID:AB_2915951; GH: Cat# DGH00; RRID:AB_2923238). Assays were performed according to manufacturers' instructions. Absorbance was measured with the BMG CLARIOstar Plus (BMG Labtech), and analyte concentrations were interpolated to the relevant standard curve using a sigmoidal, 4PL curve fitting model in GraphPad Prism (GraphPad Software). All quantitative data are presented as mean ± standard deviation.

## Results

### MAR002 binds to GHR with high affinity and durable receptor residence time

MAR002 was generated by immunizing transgenic mice with the recombinant ECD of human and cynomolgus monkey GHR protein (Methods).

Binding affinity of MAR002 to human and cynomolgus monkey GHR ECD was assessed using SPR and compared with that of the natural ligand, GH (Methods). MAR002 exhibited a binding affinity comparable to GH, with both molecules demonstrating low-picomolar affinity (Table 1).

**Table 1:** GHR binder equilibrium dissociation constants (KD) determined by SPR kinetic binding assays

	Human GHR			Cynomolgus monkey GHR			Rat GHR	Mouse GHR
GHR antagonist	*k_a_* (1/Ms)	*k_d_* (1/s)	KD (M)	*k_a_* (1/Ms)	*k_d_* (1/s)	KD (M)	KD (M)	KD (M)
Human GH	4.28E+05	1.00E-05*^a^*	2.34E-11	8.85E+05	2.89E-04	3.27E-10	ND	ND
Pegvisomant	2.58E+04	9.95E-04	3.85E-08	8.42E+04	1.02E-03	1.21E-08	ND	ND
MAR002	2.94E+05	1.00E-05*^a^*	3.40E-11	3.38E+05	1.00E-05*^a^*	2.96E-11	NB	NB

^*a*^Technical limit of detection with dissociation time of 10 minutes.

Kinetic characterization and comparison of human GH, pegvisomant, and MAR002 binding to human and cynomolgus monkey GHR as assessed by SPR. Data were buffer and reference-surface subtracted and fitted with a 1:1 Langmuir binding equation. Binding kinetic experiments were performed in 3 independent runs, with consistent results obtained. Values reported represent data from a single representative experiment. Abbreviations: GH, growth hormone; GHR, growth hormone receptor; *k_a_*, association constant; *k_d_*, dissociation constant; KD, equilibrium dissociation constant; NB, no binding; ND, not done; SPR, surface plasmon resonance.

The binding of MAR002 was next compared with pegvisomant. MAR002 exhibited exceptionally slow dissociation rates (*k_d_*) from both human and cynomolgus monkey GHR, with values approaching the technical limit of detection of the assay. This slow off-rate was the primary driver of its potent binding affinity, resulting in KD values of 3.40 × 10^−11^ M for human GHR and 2.96 × 10^−11^ M for cynomolgus monkey GHR. These affinities were ∼1000-fold greater than those of pegvisomant (KD ≈ 3.85 × 10^−8^ M and 1.21 × 10^−8^ M, respectively). In contrast, no binding was observed for MAR002 to rat or mouse GHR (Table 1), confirming the cynomolgus monkey as the appropriate pharmacologically relevant species for subsequent in vivo evaluation. Taken together, these data demonstrated that MAR002 binds GHR with high affinity and exhibits prolonged receptor residence time, suggesting the potential for sustained pharmacologic inhibition of GHR signaling. Cross-reactive binding to both human and cynomolgus monkey GHR was a key selection criterion during the discovery process.

### MAR002 demonstrates enhanced FcRn binding

MAR002 was engineered for prolonged systemic exposure through Fc region modification. The LS (M428L/N434S) mutations enhance FcRn binding at endosomal pH (∼6.0) while maintaining pH-dependent release at physiological pH (∼7.4), reducing lysosomal degradation and extending antibody half-life through more efficient FcRn-mediated recycling (16). Consistent with this design, MAR002 demonstrated increased FcRn binding at pH 6.0 relative to its precursor antibody lacking the LS mutation, with increases of 4.99-fold and 8.61-fold for human and cynomolgus monkey FcRn, respectively. MAR002 maintained appropriate pH-dependent binding selectivity, showing 155-fold weaker binding at physiological pH 7.4 compared with pH 6.0, consistent with proper FcRn-mediated recycling and antibody release at the cell surface.

### MAR002 exhibits high specificity for GHR and binds the d3-GHR isoform

Approximately 40-50% of individuals in Western populations carry at least 1 allele of the exon 3-deleted GHR (d3-GHR) isoform (20). The d3-GHR isoform lacks the 22 amino acids encoded by exon 3 of the intact GHR ECD. MAR002 bound both human and cynomolgus monkey d3-GHR isoforms with high affinity, exhibiting KD values within 1.5-fold of those observed for the respective intact GHR ECDs, a difference within the expected variability of SPR binding kinetic measurements. These data indicate that MAR002 binding is preserved for the d3-GHR isoform.

Given that GH can engage both the GHR and the PRLR, and that these receptors share conserved structural features (21), selectivity was assessed to confirm receptor specificity. No binding of MAR002 to PRLR was observed, supporting specificity for GHR.

### MAR002 potently inhibits GH-induced signaling in vitro

**Figure 1:**
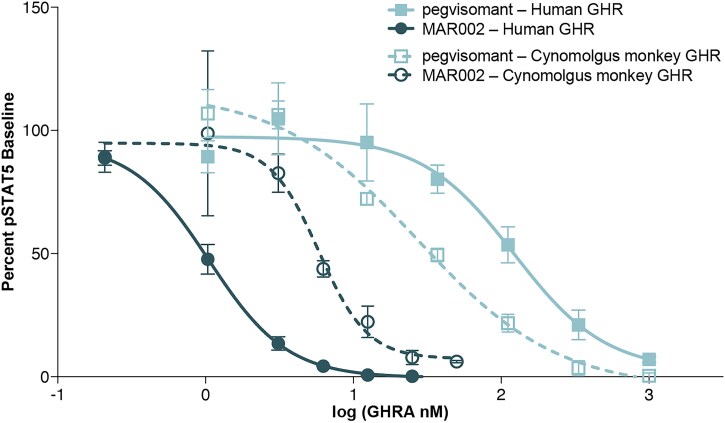
MAR002 demonstrates greater potency vs pegvisomant in inhibiting GH-induced GHR signaling. Dose-response curves show the inhibition of GH-induced STAT5 phosphorylation in HEK293 cells stably expressing full-length human or cynomolgus monkey GHR. Cells were preincubated with increasing concentrations of either MAR002 or pegvisomant prior to stimulation with 5 nM human GH. Data show pSTAT5 mean ± SD as a percentage of baseline (*n* ≥ 2). IC_50_ values derived from these dose-response curves are reported in Table 2.

We next investigated whether the high affinity, slow dissociation binding profile of MAR002 translated into enhanced functional potency. We assessed the effect of MAR002 on GH-induced STAT5 phosphorylation, a canonical downstream signaling pathway activated by GHR, in a cell-based assay. Consistent with its high binding affinity, MAR002 demonstrated potent, dose-dependent inhibition of GHR signaling (Fig. 1). For human GHR, MAR002 achieved an IC_50_ of 1.05 nM, representing a >100-fold improvement in potency as compared with pegvisomant (IC_50_ = 122 nM) (Table 2). Similarly, MAR002 demonstrated greater potency over pegvisomant for cynomolgus monkey GHR (Table 2). These results confirmed that the high binding affinity and slow off-rate of MAR002 directly translated to superior functional inhibition in vitro.

**Table 2:** MAR002 and pegvisomant IC_50_ for inhibition of GHR signaling

	IC_50_ (nM)	
	MAR002	Pegvisomant
Human GHR	1.05	122
Cynomolgus monkey GHR	5.82	27.5

Comparison of the inhibitory effects of MAR002 and pegvisomant on the phosphorylation of STAT5, a downstream readout of GHR signaling, using a cell-based assay. IC_50_ values were derived from the dose-response curves shown in Fig. 1. Assay was performed in at least duplicate. Abbreviations: GHR, growth hormone receptor; IC_50_, half-maximal inhibitory concentration; STAT5, signal transducer and activator of transcription 5.

To confirm that the high potency observed in the cell-based assay was an intrinsic property of the antigen-binding domain and not an artifact of bivalent binding (avidity) in a system with potentially high GHR density, a monovalent Fab fragment of MAR002 was generated (Methods). When tested at 30 nM, the MAR002 Fab inhibited GH-induced signaling within 1.2-fold of the full-length IgG1 format for both human and cynomolgus monkey GHR, confirming that the potency of MAR002 is driven by the high affinity of its individual binding arms and not by avidity.

### MAR002 binds a distinct allosteric epitope and mediates noncompetitive inhibition of GHR signaling

**Figure 2:**
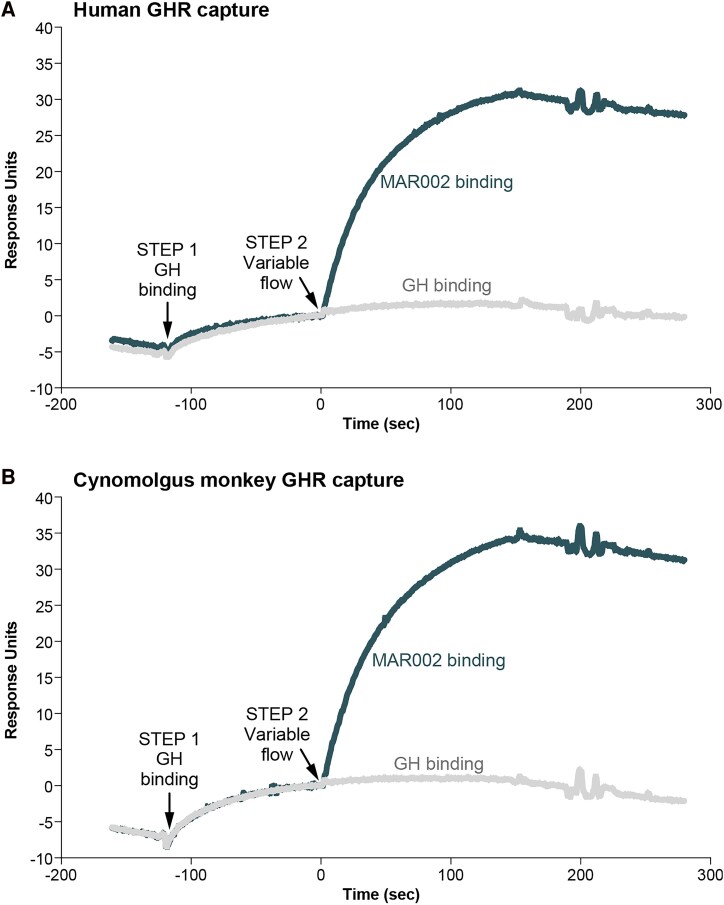
Epitope binning demonstrates noncompetitive binding of MAR002 to GHR. Representative SPR sensorgrams showing binding of MAR002 to (A) immobilized human GHR ECD and (B) immobilized cynomolgus monkey GHR ECD following preassociation with GH. The MAR002 curve shows binding to the preformed GHR-GH complex, while the GH control curve represents a second injection of GH administered following initial GHR-GH complex formation. These data demonstrate that MAR002 binds to the GHR-GH complex, indicating simultaneous binding. Assay was performed in duplicate.

To elucidate the mechanism underlying the potent MAR002-induced inhibition of GH signaling, we first performed SPR epitope binning experiments. These assays were carried out using both human and cynomolgus monkey GHR ECD. Results showed that MAR002 and GH can bind to GHR simultaneously without displacing one another (Fig. 2), providing evidence consistent with a noncompetitive, allosteric mechanism of inhibition.

**Figure 3:**
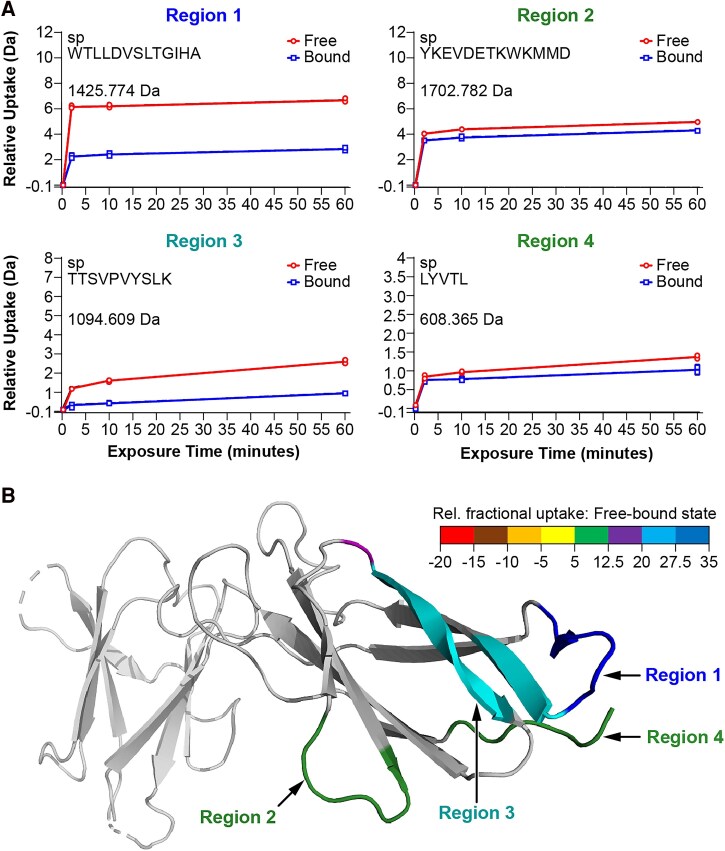
MAR002 binds to a distinct allosteric epitope on the human GHR ECD. (A) Deuterium uptake plots from HDX-MS analysis of 4 protected peptide regions, showing reduced deuterium incorporation in the MAR002-bound state (square) compared with the free GHR state (circle). Note the asparagine residues at N-linked glycosylation sites are represented as aspartate due to deamidation by PNGase Rc during deglycosylation. (B) Mapping of the 4 peptide regions protected by MAR002 binding onto a surface representation of a single GHR ECD monomer (PDB: 3HHR), showing a distributed conformational epitope that is spatially distinct from the known GH binding interface and is proximal to the GHR dimerization interface.

To provide a structural basis for this noncompetitive mechanism, we next identified the MAR002 binding site using HDX-MS. The analysis revealed 4 distinct regions on the GHR ECD that were significantly protected from deuterium incorporation upon MAR002 binding: region 1, amino acids 158-169 (TLLNVSLTGIHA); region 2, 198-204 (EVNETKW); region 3, 212-221 (TTSVPVYSLK); and region 4, 249-251 (VTL) (Fig. 3A). Mapping these regions onto the GHR crystal structure (Fig. 3B) confirmed that the epitope is spatially distinct from the canonical GH binding sites (22), providing structural support for an allosteric mechanism of inhibition. Notably, the 2 regions with the highest magnitude of protection (region 1: 158-169 and region 3: 212-221) are proximal to the dimerization interface of GHR, suggesting that MAR002 binding potentially interferes with receptor dimerization required for signal transduction.

**Figure 4:**
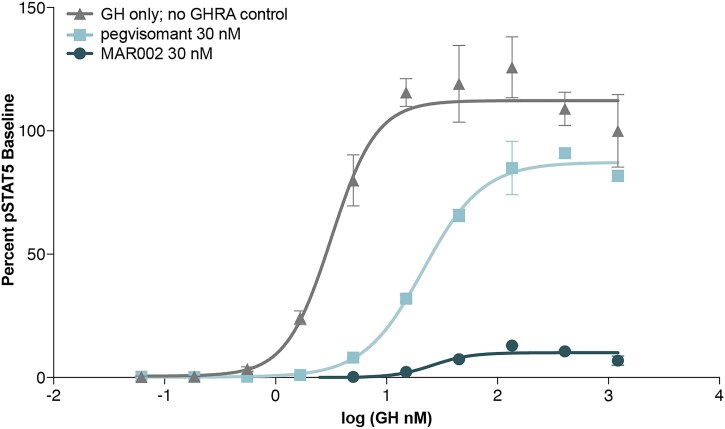
The allosteric mechanism of MAR002 confers robust inhibition that is not overcome by high GH concentrations. Inhibition of GH-induced STAT5 phosphorylation in the presence of increasing concentrations of human GH. HEK293 cells stably expressing full-length human GHR were incubated with a fixed 30 nM concentration of MAR002 or pegvisomant and challenged with human GH ranging from 0.06 to 1215 nM. MAR002 maintains inhibition of GHR signaling in the presence of increasing GH concentrations, including supraphysiological levels, whereas pegvisomant activity is competitively reduced. Data show pSTAT5 mean ± SD as a percentage of baseline (*n* ≥ 2). Note: the x-axis represents log_10_ GH concentration in nM; the maximum GH concentration tested was 1215 nM.

Based on these findings, we hypothesized that MAR002 would maintain its inhibitory effect on GH-induced STAT5 phosphorylation, even when challenged with excess GH, a condition relevant to the clinical setting. To test this, we performed a cell-based functional assay to determine the inhibitory effects of MAR002 and pegvisomant on GH-induced STAT5 phosphorylation in the presence of increasing concentrations of GH. As shown in Fig. 4, the inhibitory effect of the competitive antagonist, pegvisomant, was progressively overcome as GH concentrations increased. In contrast, MAR002 maintained stable and near-complete suppression of GHR signaling (>90%), even at supraphysiological GH concentrations up to 1215 nM, well exceeding GH levels reported in uncontrolled acromegaly (1, 4). These data demonstrate that MAR002-mediated inhibition is not overcome by high levels of GH, a key functional advantage conferred by its allosteric mechanism.

### MAR002 demonstrates prolonged IGF-1 suppression in cynomolgus monkeys

Finally, we sought to determine whether the potent in vitro properties of MAR002 would translate to prolonged in vivo efficacy. The cynomolgus monkey was selected as the appropriate PD model, as it is a well-established model for GHR-targeted therapeutics and has conserved GH–IGF-1 physiology with humans (7). The cynomolgus monkey GHR shares 94.8% amino acid sequence identity with the human GHR (accession numbers: human GHR P10912; cynomolgus monkey GHR EHH62357; NCBI Taxonomy IDs 9606 and 9541, respectively), supporting the translational relevance of this model. This selection was further validated by the in vitro binding and functional assays described above, which demonstrated potent binding and functional activity of MAR002 on the cynomolgus monkey GHR. The absence of MAR002 binding to either rat or mouse GHR (Table 1) confirmed that rodents are not a pharmacologically relevant species for in vivo evaluation of this molecule.

**Figure 5:**
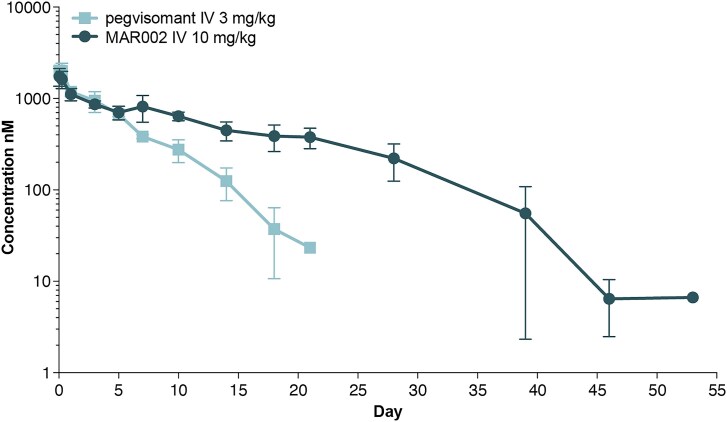
MAR002 exhibits an extended PK profile in cynomolgus monkeys. Mean serum concentration-time profiles of MAR002 and pegvisomant following a single IV dose in male cynomolgus monkeys (mean ± SD; *n* = 3 animals per group). Animals received either 10 mg/kg of MAR002 or an equimolar 3 mg/kg dose of pegvisomant.

In a head-to-head comparison study, cynomolgus monkeys (*n* = 3 per group) received either a single IV dose of MAR002 (10 mg/kg) or an equimolar dose of pegvisomant (3 mg/kg), which produced comparable maximal suppression of circulating IGF-1. MAR002 exhibited a favorable pharmacokinetic profile with sustained serum concentrations, consistent with its engineered half-life extension (Fig. 5).

**Figure 6:**
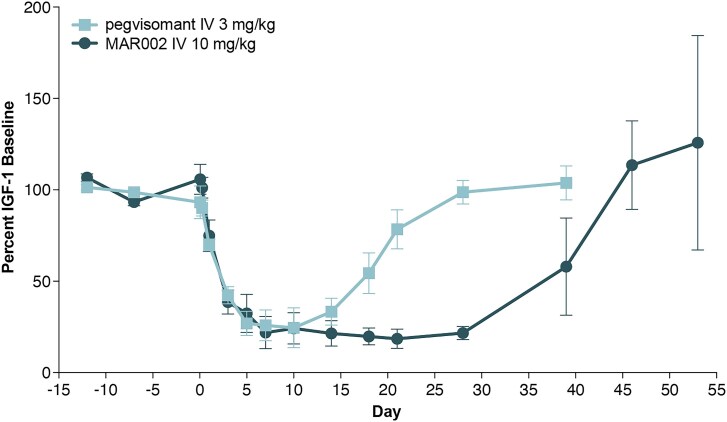
A single dose of MAR002 results in durable and prolonged IGF-1 suppression in vivo. Mean percent change in serum IGF-1 levels from baseline in cynomolgus monkeys following a single IV dose of MAR002 (10 mg/kg) or equimolar dose of pegvisomant (3 mg/kg). MAR002 maintained ≥50% suppression of circulating IGF-1 for 36 days, compared with 15 days for pegvisomant. Baseline serum IGF-1 concentration was defined as the mean of 2 predose samples (Day −12 and Day −7), which are shown to illustrate baseline variability. Postdose IGF-1 values were expressed as a percentage of baseline (mean ± SD; *n* = 3 animals per group).

In line with these PK observations, MAR002 demonstrated an extended PD effect, maintaining robust IGF-1 suppression (≥50%) for 36 days (Fig. 6; Table S1; 23). In contrast, the effect of pegvisomant was far less durable, with IGF-1 suppression lasting only 15 days (Fig. 6; Table S1; 23). IGF-1 suppression was consistent across individual animals in the MAR002 treatment group. This >2-fold increase in duration of action provides strong preclinical support for MAR002 as a long-acting GHRA. Concurrent with IGF-1 suppression, a marked and sustained increase in serum GH was observed in MAR002-treated animals (Fig. S1; 23). This elevation in GH may reflect a combination of an increase in GH secretion due to loss of feedback-inhibition on pituitary GH secretion, driven by reduced IGF-1 levels (24), and prolonged half-life of circulating GH/GH binding protein (GHBP) complexes bound to MAR002.

## Discussion

Here, we describe the discovery and preclinical characterization of MAR002, a novel, allosteric, half-life extended monoclonal antibody antagonist of GHR. MAR002 demonstrates several advantages over pegvisomant, the current standard of care GHRA, including a distinct allosteric mechanism of inhibition, activity that is maintained at high GH concentrations, and markedly prolonged duration of action in nonhuman primates. These attributes position MAR002 as a promising long-acting therapy for the treatment of acromegaly, with the potential to reduce treatment burden while achieving more consistent IGF-1 suppression, ultimately leading to improved patient outcomes.

Many patients with acromegaly fail to achieve biochemical control with first-line SRLs, which normalize IGF-1 in only 30-50% of patients (3). Pegvisomant, the only approved GHRA, achieves IGF-1 normalization in the vast majority of patients in clinical trials, but has more limited real-world effectiveness (12). Normalization of serum IGF-1 is the primary biochemical endpoint in acromegaly. MAR002 was engineered to build upon the established efficacy of GHR antagonism while addressing the primary limitation of current therapy, namely treatment burden, through greater potency and an extended half-life. These findings support clinical development of MAR002 as both monotherapy and combination therapy.

A key differentiator of MAR002 is its allosteric mechanism of GHR inhibition, which was confirmed through a combination of functional and structural evidence. In contrast to pegvisomant, which competes directly with GH for binding to GHR (7), epitope binning and HDX-MS assays indicate that MAR002 binds to a distinct site that is proximal to the GHR dimerization interface. This noncompetitive mechanism could confer a clinical advantage, particularly in patients with the highest disease burden. Clinical studies have identified that patients with high baseline IGF-1 levels are at greater risk for pegvisomant resistance (25, 26), a finding explained by the need for higher circulating pegvisomant concentrations to overcome the greater GH secretory burden in these patients (27). Furthermore, pegvisomant treatment itself can lead to a compensatory increase in endogenous GH secretion, which could cause a feed-forward exacerbation of this competitive burden thus leading to resistance (9). These in vitro data support this concept, showing that while the inhibitory effect of pegvisomant waned as GH concentrations increased, inhibition by MAR002 was maintained at all concentrations of GH. These findings support a mechanism in which MAR002 disrupts receptor activation downstream of, and without preventing, ligand binding, highlighting an alternative strategy for targeting the GH–GHR axis through allosteric inhibition rather than direct competition. The localization of the MAR002 epitope proximal to the GHR dimerization interface provides a structural basis for this effect. GHR signaling requires Janus kinase 2 transactivation and subsequent STAT5 phosphorylation, a process that is dependent on precise receptor geometry at the dimerization interface; whether through ligand-induced dimerization or through a GH-induced conformational change within a preformed receptor dimer (7, 28). Binding of MAR002 at this site may interfere with the receptor geometry or conformational changes required for signaling complex assembly, independently of ligand occupancy.

To ensure broad applicability and safety, we confirmed 2 critical attributes. First, MAR002 binds effectively to the d3-GHR isoform, a common genetic variant present in up to 50% of some populations (20), supporting preserved receptor engagement across the patient population. Second, MAR002 is highly selective for GHR and does not cross-react with the related PRLR, a critical safety feature that mitigates the risk of off-target signaling (21).

MAR002 was specifically engineered with Fc modifications to optimize its clinical profile. The LS mutations enhanced FcRn binding, extending plasma half-life and driving the prolonged PD effect observed in vivo, while the LALA mutations abrogated unwanted effector functions to improve the safety profile (14-16).

These strong in vitro properties translated into a markedly prolonged period of IGF-1 suppression in vivo. In cynomolgus monkeys, a single dose of MAR002 maintained over 50% IGF-1 suppression for 36 days, more than twice the duration achieved by an equimolar dose of pegvisomant. Although group sizes were small, the magnitude and consistency of IGF-1 suppression across individual animals supported a clear PD effect. The concomitant rise in serum GH levels is consistent with on-target GHR antagonism due to interruption of the negative feedback somatotropic axis (24), though it is likely amplified by a PK effect whereby MAR002 extends the half-life of circulating GH/GHBP complex via a ternary complex.

We acknowledge several limitations in the present study. First, in vivo data were generated in healthy nonhuman primates; as MAR002 does not cross-react with rodent GHR, established transgenic acromegaly models could not be used. Although the high sequence homology of the GHR makes the cynomolgus monkey a highly relevant translational model, its use in healthy animals may not fully replicate the chronic supraphysiological GH secretion characteristic of acromegaly. Therefore, the ultimate efficacy and safety profile of MAR002 must be confirmed in human clinical trials. Additionally, this foundational study evaluated only a single IV administration in a small cohort of male animals. Future studies will be essential to characterize the PK/PD profile following subcutaneous administration (the intended clinical route), to assess dose-dependent responses, and to evaluate long-term safety and efficacy under a repeat-dosing regimen in both sexes. Despite these limitations, the magnitude and consistency of the PD response, together with the robust in vitro dataset, provide strong preclinical proof-of-concept for MAR002 as a long-acting GHRA.

The therapeutic landscape for acromegaly is evolving, with the development of novel somatostatin receptor ligand therapeutics that improve upon current options, including depot formulations that reduce injection frequency, subcutaneous dosing options that improve injection tolerability, and oral formulations that avoid injections entirely (2, 8, 29). In addition to SRLs, GHR antagonism with pegvisomant provides an effective strategy for biochemical control and is used both as monotherapy and in combination with SRLs in patients requiring enhanced biochemical control (2, 30). Within this therapeutic context, MAR002 represents a next-generation and mechanistically distinct GHRA developed to improve upon the established efficacy of pegvisomant while addressing treatment burden for patients. Its allosteric mechanism, potent inhibition of GHR signaling, and the extended half-life profile collectively support the potential for more potent GHR blockade, reduced dosing frequency, and improved treatment adherence for patients. Together, these findings support the continued clinical development of MAR002 as a long-acting GHRA for acromegaly. A first-in-human, randomized, placebo-controlled Phase 1 study to evaluate the safety, tolerability, and PK/PD of MAR002 in healthy volunteers is underway (ClinicalTrials.gov Identifier: NCT07195175).

## Data Availability

The data that support the findings of this study are available from the corresponding author upon reasonable request.

## Abbreviations

ECD: extracellular domain
ELISA: enzyme-linked immunosorbent assay
FACS: fluorescence-activated cell sorting
FcRn: neonatal Fc receptor
GH: growth hormone
GHR: growth hormone receptor
GHRA: growth hormone receptor antagonist
GLP: good laboratory practice
HDX-MS: hydrogen–deuterium exchange mass spectrometry
HTRF: homogeneous time-resolved fluorescence
IC_50_: half-maximal inhibitory concentration
IGF-1: insulin-like growth factor-1
IV: intravenous
*k_a_*: association constant
*k_d_*: dissociation constant
kDa: kilodalton
KD: equilibrium dissociation constant
NB: no binding
ND: not done
PD: pharmacodynamics
PDB: protein data bank
PK: pharmacokinetics
SD: standard deviation
pSTAT5: phosphorylated Signal Transducer and Activator of Transcription 5
SRLs: somatostatin receptor ligands
SPR: surface plasmon resonance
STAT5: signal transducer and activator of transcription 5

## References

[1] Katznelson L, Laws ER Jr, Melmed S, et al Acromegaly: an endocrine society clinical practice guideline. J Clin Endocrinol Metab. 2014;99(11):3933‐3951. 25356808 10.1210/jc.2014-2700

[2] Melmed S, di Filippo L, Fleseriu M, et al Consensus on acromegaly therapeutic outcomes: an update. Nat Rev Endocrinol. 2025;21(11):718‐737. 40804505 10.1038/s41574-025-01148-2

[3] Carmichael JD, Bonert VS, Nuño M, Ly D, Melmed S. Acromegaly clinical trial methodology impact on reported biochemical efficacy rates of somatostatin receptor ligand treatments: a meta-analysis. J Clin Endocrinol Metab. 2014;99(5):1825‐1833. 24606084 10.1210/jc.2013-3757 PMC4010703

[4] Ershadinia N, Tritos NA. Diagnosis and treatment of acromegaly: an update. Mayo Clin Proc. 2022;97(2):333‐346. 35120696 10.1016/j.mayocp.2021.11.007

[5] Giustina A, Mazziotti G, Torri V, Spinello M, Floriani I, Melmed S. Meta-analysis on the effects of octreotide on tumor mass in acromegaly. PLoS One. 2012;7(5):e36411. 22574156 10.1371/journal.pone.0036411 PMC3344864

[6] Maia B, Kasuki L, Gadelha MR. Novel therapies for acromegaly. Endocr Connect. 2020;9(12):R274‐R285. 33434151 10.1530/EC-20-0433 PMC7774757

[7] Kopchick JJ, Parkinson C, Stevens EC, Trainer PJ. Growth hormone receptor antagonists: discovery, development, and use in patients with acromegaly. Endocr Rev. 2002;23(5):623‐646. 12372843 10.1210/er.2001-0022

[8] Melmed S . Pituitary-tumor endocrinopathies. N Engl J Med. 2020;382(10):937‐950. 32130815 10.1056/NEJMra1810772

[9] Trainer PJ, Drake WM, Katznelson L, et al Treatment of acromegaly with the growth hormone-receptor antagonist pegvisomant. N Engl J Med. 2000;342(16):1171‐1177. 10770982 10.1056/NEJM200004203421604

[10] van der Lely AJ, Hutson RK, Trainer PJ, et al Long-term treatment of acromegaly with pegvisomant, a growth hormone receptor antagonist. Lancet. 2001;358(9295):1754‐1759. 11734231 10.1016/s0140-6736(01)06844-1

[11] Camara R, Venegas E, Garcia-Arnes JA, et al Treatment adherence to pegvisomant in patients with acromegaly in Spain: PEGASO study. Pituitary. 2019;22(2):137‐145. 30756345 10.1007/s11102-019-00943-1

[12] Fleseriu M, Fuhrer-Sakel D, van der Lely AJ, et al More than a decade of real-world experience of pegvisomant for acromegaly: ACROSTUDY. Eur J Endocrinol. 2021;185(4):525‐538. 34342594 10.1530/EJE-21-0239 PMC8428076

[13] Yuen KCJ, Vila G, Bernabeu I, et al Long-term safety and efficacy of pegvisomant monotherapy for acromegaly: final data from the full ACROSTUDY cohort. Pituitary. 2026;29(3):71. 41984268 10.1007/s11102-026-01673-x PMC13083532

[14] Hale G . Living in LALA land? Forty years of attenuating Fc effector functions. Immunol Rev. 2024;328(1):422‐437. 39158044 10.1111/imr.13379 PMC11659930

[15] Hale G, De Vos J, Davy AD, Sandra K, Wilkinson I. Systematic analysis of Fc mutations designed to reduce binding to Fc-gamma receptors. MAbs. 2024;16(1):2402701. 39279104 10.1080/19420862.2024.2402701 PMC11407402

[16] Zalevsky J, Chamberlain AK, Horton HM, et al Enhanced antibody half-life improves in vivo activity. Nat Biotechnol. 2010;28(2):157‐159. 20081867 10.1038/nbt.1601 PMC2855492

[17] National Research Council (NRC) . Guide for the Care and Use of Laboratory Animals. 8th ed. 2011.

[18] Veldhuis JD, Roemmich JN, Richmond EJ, Bowers CY. Somatotropic and gonadotropic axes linkages in infancy, childhood, and the puberty-adult transition. Endocr Rev. 2006;27(2):101‐140. 16434512 10.1210/er.2005-0006

[19] Waxman DJ, O'Connor C. Growth hormone regulation of sex-dependent liver gene expression. Mol Endocrinol. 2006;20(11):2613‐2629. 16543404 10.1210/me.2006-0007

[20] Bernabeu I, Alvarez-Escola C, Quinteiro C, et al The exon 3-deleted growth hormone receptor is associated with better response to pegvisomant therapy in acromegaly. J Clin Endocrinol Metab. 2010;95(1):222‐229. 19850678 10.1210/jc.2009-1630

[21] Kelly PA, Djiane J, Postel-Vinay MC, Edery M. The prolactin/growth hormone receptor family. Endocr Rev. 1991;12(3):235‐251. 1935820 10.1210/edrv-12-3-235

[22] de Vos AM, Ultsch M, Kossiakoff AA. Human growth hormone and extracellular domain of its receptor: crystal structure of the complex. Science. 1992;255(5042):306‐312. 1549776 10.1126/science.1549776

[23] Kurylo K, Bouchard PR, Milton MN, et al 2026. Serum IGF-1 and GH pharmacodynamic data in cynomolgus monkeys following a single intravenous dose of MAR002. Figshare. 10.6084/m9.figshare.33131693.

[24] Melmed S . Medical progress: acromegaly. N Engl J Med. 2006;355(24):2558‐2573. 17167139 10.1056/NEJMra062453

[25] Giampietro A, Chiloiro S, Urbani C, et al Factors associated with disease control failure in acromegaly patients treated with pegvisomant: an ACROSTUDY analysis. Endocr Connect. 2024;13(3):e230247. 38197875 10.1530/EC-23-0247 PMC10895310

[26] Ragonese M, Grottoli S, Maffei P, et al How to improve effectiveness of pegvisomant treatment in acromegalic patients. J Endocrinol Invest. 2018;41(5):575‐581. 29080965 10.1007/s40618-017-0773-0

[27] Madsen M, Fisker S, Feldt-Rasmussen U, et al Circulating levels of pegvisomant and endogenous growth hormone during prolonged pegvisomant therapy in patients with acromegaly. Clin Endocrinol (Oxf). 2014;80(1):92‐100. 23650996 10.1111/cen.12239

[28] Brooks AJ, Dai W, O'Mara ML, et al Mechanism of activation of protein kinase JAK2 by the growth hormone receptor. Science. 2014;344(6185):1249783. 24833397 10.1126/science.1249783

[29] Fleseriu M, Langlois F, Lim DST, Varlamov EV, Melmed S. Acromegaly: pathogenesis, diagnosis, and management. Lancet Diabetes Endocrinol. 2022;10(11):804‐826. 36209758 10.1016/S2213-8587(22)00244-3

[30] Melmed S, Bronstein MD, Chanson P, et al A consensus statement on acromegaly therapeutic outcomes. Nat Rev Endocrinol. 2018;14(9):552‐561. 30050156 10.1038/s41574-018-0058-5 PMC7136157

